# Trends and quality of randomized controlled trials on acupuncture conducted in Japan by decade from the 1960s to the 2010s: a systematic review

**DOI:** 10.1186/s12906-023-03910-3

**Published:** 2023-03-27

**Authors:** Shoko Masuyama, Hitoshi Yamashita

**Affiliations:** Graduate School of Health Sciences, Morinomiya University of Medical Sciences, 1-26-16 Nanko-Kita, Suminoe-Ku, Osaka City, 559-8611 Japan

**Keywords:** Randomized controlled trial, Acupuncture, Japan, Quality, Systematic review, Decade-wise trends

## Abstract

**Background:**

More new randomized controlled trials (RCTs) on acupuncture have been published in Japan since our last updated systematic review (2010). This systematic review aimed to evaluate the quality of RCTs on acupuncture conducted in Japan and understand the decade-wise changes in the methodological characteristics of the relevant RCTs.

**Methods:**

The literature search was performed using Ichushi Web, the Cochrane Central Register of Controlled Trials (CENTRAL), PubMed and our team’s compilation of relevant papers. We included full-length papers reporting RCTs that examined the clinical effects of acupuncture on patients in Japan published in or before 2019. We assessed the risk of bias (RoB), sample size, control setting, negative trial reporting, informed consent, ethics approval, trial registration, and adverse event reporting.

**Results:**

A total of 99 articles reporting 108 eligible RCTs were identified. The number of RCTs published in each decade was 1, 6, 9, 5, 40, and 47 in the 1960s, 1970s, 1980s, 1990s, 2000s, and 2010s, respectively. Quality assessment using the Cochrane RoB tool revealed that “sequence generation” improved in and after 1990 (73%–80% of RCTs were rated as “low”) and “blinding of outcome assessors” slightly improved in and after the 2000s (40%–50% judged as “low”). However, “high” or “unclear” remained the dominant grades in other domains. Clinical trial registration and adverse events were reported only in 9% and 28% of the included RCTs even in the 2010s, respectively. A different acupuncture method or different point selection (e.g., deep vs. shallow insertion) was the most dominant control setting before 1990, while sham (or “placebo”) needling and/or sham acupoints became the most dominant in the 2000s. The proportion of RCTs with positive results was 80% in the 2000s and 69% in the 2010s.

**Conclusions:**

The quality of RCTs on acupuncture conducted in Japan did not appear to have improved over the decades except for “sequence generation.” While the culture of submitting negative trial reports was prevalent in the Japanese acupuncture research milieu as late as the 1990s, the overall quality of the relevant trials needs to be further improved.

**Supplementary Information:**

The online version contains supplementary material available at 10.1186/s12906-023-03910-3.

## Introduction

Evidence-based medicine (EBM) has been a common concept in the field of healthcare worldwide. According to the concept of EBM, a rigorously performed randomized controlled trial (RCT) is a study design that provides the methodologically strongest evidence for evaluation of the clinical effectiveness of healthcare interventions [1]. The critical appraisal of acupuncture is no exception. A rough search in the PubMed database for RCTs whose title includes the word “acupuncture” yields more than 2,500 articles published before 2020. Examination of the decade-wise quantitative changes in RCTs shows that 18, 46, 183, 816, and 1,487 papers were published in the 1970s, 1980s, 1990s, 2000s, and 2010, respectively (searched with PubMed on October 23, 2021). Although these are the results of a crude preliminary search and some RCTs did not evaluate the clinical effect of acupuncture, the number of RCTs on acupuncture has obviously increased remarkably.

Thus, similar to other fields of healthcare, the evidence-based approach of performing RCTs to verify the effectiveness of acupuncture seems to have progressed since the late twentieth century. However, the quality of the RCTs is not always high, cautioning against interpreting the results of RCTs with unwarranted confidence [1]. There is scope for improvement in the overall quality of RCTs on acupuncture with respect to the risk of bias (RoB) [2]. This is also true for clinical trials on acupuncture conducted in Japan at least before 2008, since their quality is not necessarily high [3, 4].

In our previous systematic reviews [3, 4], we included not only RCTs but also non-randomized controlled clinical trials and RCTs that were reported only as conference abstracts. Therefore, detailed assessment of the quality of acupuncture RCTs conducted in Japan was difficult. Furthermore, we used the Jadad score [5] to evaluate the quality of the controlled clinical trials included in the reviews. This 5-point score is simple and useful for assessing the appropriateness of randomization, blinding, and drop-out reporting, but we could not examine allocation concealment, management of incomplete outcome data, and selective outcome reporting. Moreover, we did not assess whether or not informed consent, ethics approval, trial registration, and adverse events were reported, which are factors that may indicate the quality of each trial. More RCTs on acupuncture were published in Japan, since our last updated systematic review, which was conducted 12 years ago [4]. Moreover, there has been a complete shift in the standard assessment instrument to the Cochrane Collaboration’s RoB tool [6].

Accordingly, in this updated systematic review, we included only the relevant articles other than conference abstracts and used the Cochrane RoB assessment tool to evaluate the quality of RCTs on acupuncture conducted in Japan. Furthermore, this study aimed to analyze and understand the decade-wise changes in the characteristics of the relevant RCTs, focusing on factors such as number, magnitude, quality, conductor, and control setting.

## Methods

This study did not meet the inclusion criteria for the International Prospective Register of Systematic Reviews (PROSPERO) [7] because it focused on changes in the quality and methodological characteristics of Japanese RCTs on acupuncture grouped by decade. Therefore, this study protocol was not registered with PROSPERO.

### Search methods

The database search was performed between July 6 and July 26, 2021. We used Ichushi Web, and the Cochrane Central Register of Controlled Trials (CENTRAL) for the literature search. Ichushi Web is the largest database of Japanese medical literature that contains information on approximately 14 million articles [8], and the CENTRAL is a highly concentrated source of reports of randomized and quasi-randomized controlled trials derived from bibliographic databases of PubMed and Embase, and other published and unpublished sources including CINAHL, ClinicalTrials.gov and the World Health Organization (WHO)’s International Clinical Trials Registry Platform [9]. On January 22, 2023, we performed an additional search of PubMed. The year of publication was limited to 2019 or earlier.

For the Ichushi Web search, we used Japanese keywords and thesaurus terms translating to acupuncture, electroacupuncture, meridian, and acupoint. We limited the search to articles that were classified as clinical trials, RCTs, quasi-RCTs, controlled trials, or crossover trials. We did not include reports classified as conference abstracts. For the CENTRAL search, we used MeSH terms such as “acupuncture therapy,” “acupuncture,” “moxibustion,” “meridians,” “acupuncture points,” and “dry needling,” and keywords such as “Acupunct*,” “moxibust*,” “moxa,” “electroacupunct*,” “dry NEXT needl*,” “acupoint*,” and “acupuncture point*.” We used the following MeSH terms for the PubMed search, which was limited to randomized controlled trials: “acupuncture therapy,” “moxibustion,” “meridians,” “acupuncture points,” and keywords such as “dry needl*” and “Japan[Affiliation].” The details of the search strategy are presented in Additional file 1.

We also used our own compilations of articles on clinical trials on acupuncture published in Japan for nearly 30 years. This record contained some eligible RCTs that were not listed in or hit by the above-mentioned databases. We collected these RCTs through a weekly PubMed/Medline search using only one keyword of “acupuncture” since 1992, periodical browsing of major domestic acupuncture journals, a list called the “Japanese Acupuncture RCT/CCT Abstract Table” on the internet [10], The Japan Society of Acupuncture and Moxibustion’s database “Japanese Acupuncture Comprehensive Literature Database” [11], and our previous systematic reviews [3, 4].

### Study selection

The inclusion criteria for the RCTs were as follows: (1) studies with a date of publication in 2019 or earlier; (2) those conducted in Japan; (3) those whose language of publication was Japanese or English; (4) studies reported as a full-length article, short report, proceedings, or letter to the editor; (5) studies that entailed acupuncture treatment in which needles were inserted into the skin, including press tack needles, and intradermal needles; and (6) studies that examined the clinical effects of acupuncture in patients.

We also included studies that recruited healthy participants if the outcome measure was fatigue, obesity, neck-shoulder stiffness (*Katakori* in Japanese), asthenopia, acne, postpartum conditions, leg edema, or sensitivity to cold (*Hiesho* in Japanese) because these participants were in fact “patients” who were originally symptomatic, even if the authors of those studies stated that they recruited “healthy” participants. We also included crossover RCTs. If the method of randomization did not entail random allocation, we included only those studies wherein the authors clearly described that they performed “randomization.”

The exclusion criteria for the RCTs were as follows: (1) studies that were reported only as conference abstracts; (2) studies on the effects of moxibustion, laser acupuncture (low-level laser irradiation therapy), blood-letting acupuncture, microcone stimulation, and pressing/rubbing with non-insertive pediatric acupuncture device or spoon needle; (3) studies that included healthy participants or athletes in whom symptoms such as muscle soreness were induced intentionally; and 4) n-of-1 trials.

We excluded RCTs that were reported only as conference abstracts because the information was very scarce to assess the quality of the trial.

### Data screening

Two reviewers (SM and HY) independently screened the papers yielded by the database searches. First, the papers were screened by title and abstract, followed by perusal of the full text. Duplicate publications were eliminated after reading the full text. Subsequently, the reviewers collated their screening results, discussed the discrepancies in selection/exclusion, and reached an agreement.

### Data extraction

We extracted information on the first author, title, year of publication, journal name, field of the journal, language, condition treated, affiliation of the first author, trial design, sample size, acupuncture stimulation method, type of control group, results of the main outcome, informed consent, ethics committee approval, trial registration, and adverse events from the studies included in the review. We input these data into Microsoft Excel to achieve aggregation by decade.

### Assessment of RoB and other aspects of quality

Two independent reviewers (SM and HY) examined the RoB of each RCT using the Cochrane Collaboration’s RoB assessment tool [6]. While the domains of the first version of the RoB assessment tool focused on a specific method (e.g., “blinding of participants”) [6], those of the updated tool (i.e., RoB 2) explicitly refer to bias itself (e.g., “bias due to deviations from intended interventions”) [12]. We used the first version of the Cochrane RoB tool [6] to compare our data with RCTs conducted in other countries based on a similar study [2].

We examined the following six domains [6] in this review.

1. Sequence generation: “Was the allocation sequence adequately generated?”
2. Allocation concealment: “Was allocation adequately concealed?”
3. Blinding of participants, personnel, and outcome assessors: “Was knowledge of the allocated intervention adequately prevented during the study?” (The RoB for blinding of patients, therapists, and outcome assessors was judged separately.)
4. Incomplete outcome data: “Were incomplete outcome data adequately addressed?”
5. Selective outcome reporting: “Are reports of the study free of suggestion of selective outcome reporting?”
6. Other sources of bias: “Was the study apparently free of other problems that could put it at a high risk of bias?”

The two reviewers read through the papers reporting the RCTs, and independently judged the RoB to be high, low, or unclear for each domain. Both reviewers followed the criteria for judging RoB described in the Cochrane Handbook for Systematic Reviews of Interventions [6]. After independent judgment, all disagreements were resolved by discussion between the two reviewers.

We also assessed other indices: sample size, in order to determine the magnitude of the included RCTs, and whether informed consent, ethics approval, trial registration, and adverse events were reported, in order to estimate the quality of the trials that could not be assessed using the Cochrane RoB assessment tool.

We compiled and compared the above-mentioned data and other key characteristics of the included RCTs, which were grouped by decade.

## Results

### Characteristics of the RCTs grouped by decade

**Fig. 1:**
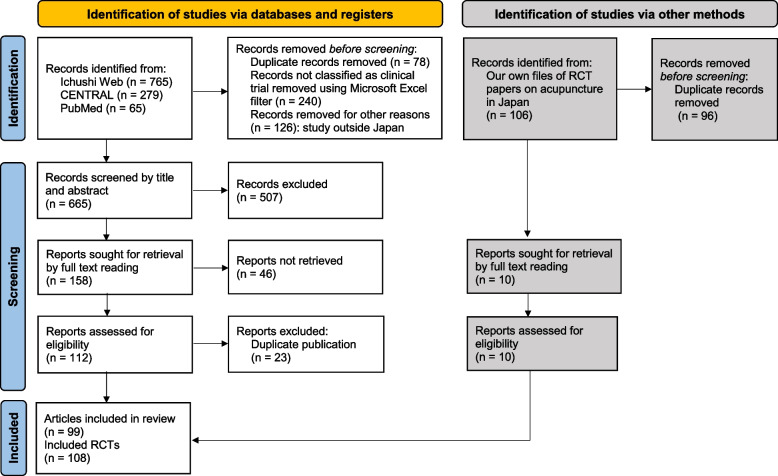
Flow diagram of literature selection (modified from the PRISMA 2020 flow diagram template for systematic reviews [13])

As shown in Fig. 1 (modified from the PRISMA 2020 flow diagram template for systematic reviews [13]), a total of 99 articles reporting 108 eligible RCTs were identified through the literature screening process (see Additional file 2 for a list of all the articles reporting the included RCTs published in Japanese or English). The studies were published between 1969 and 2019, as we did not include those published in and after 2020.

Table 1 depicts the principal characteristics of included RCTs stratified by decade. The first acupuncture RCT conducted in Japan was published in 1969 [14]; this trial compared deep and shallow needling for sciatica, and the sample size was 32. The number of RCTs increased in each subsequent decade, except for the 1990s. Overall, 81 (75%) of the 108 RCTs were reported in Japanese. Before the 2000s, all RCTs were published in Japanese in domestic journals, and 14 RCTs (36%) in the 2000s and 13 (27%) in the 2010s were published in English. We found that 86% (93 of 108) of the RCTs were reported in journals in the field of acupuncture or complementary and alternative medicine.

**Table 1:** Characteristics of RCTs conducted in Japan on acupuncture for patients

**Decade**	**Number of RCTs**	**Language**	**Journal type**	**Condition treated**	**Method of stimulation**	**Point selection**	**Number of arms**	**Crossover trial**
		** Japanese/English**	**domestic/international** **acupuncture/CAM/CWM**	** musculoskeletal/others**	** manual needling/** **electroacupuncture/press tack needle** **/intradermal needle/others** (Some data duplicated)	**body acupoint** **/auricular point** **/trigger point** **/tender point** **/others** (Some data duplicated)	** 2/3/4/5**	**yes/no**
1960s	1	1/0	1/0 1/0/0	1/0	1/0/0/0/0	1/0/0/0/0	1/0/0/0	0/1
1970s	6	6/0	6/0 6/0/0	5/1	6/0/0/0/0	3/0/0/0/3	6/0/0/0	0/6
1980s	9	9/0	9/0 9/0/0	3/6	2/2/0/5/0	1/5/0/1/2	8/1/0/0	2/7
1990s	5	5/0	5/0 4/0/1	4/1	3/1/0/0/0 (1 unknown: no relevant information provided)	3/0/0/0/1 (1 unknown: no relevant information provided)	3/0/1/1	1/4
2000s	40	26/14	26/14 29/5/6	28/12	29/10/3/5/1	27/1/8/12/3	29/6/5/0	6/34
2010s	47	34/13	36/11 24/16/8	14/33	36/10/5/0/0	35/0/4/4/14	35/11/1/0	7/40
Total	108	81/27	83/25 72/21/15	55/53	77/22/8/10/1	70/6/12/17/23	82/18/7/1	16/92

*RCT* Randomized controlled trial, *CAM* Complementary and alternative medicine, *CWM* Conventional Western medicine

More than 50% of the RCTs in this review included treatment of musculoskeletal conditions, although this trend did not hold true for the 1980s and 2010s. Body acupuncture with manual needling (including needle retention) was the most common method of acupuncture stimulation in every decade, except for the 1980s when one research team published a series of RCTs on auricular intradermal needle acupuncture for obesity. Apropos point selection, body acupoints (non-auricular points) were the most common in every decade, except for the 1980s when a series of RCTs were published on auricular intradermal needle acupuncture for obesity.

**Fig. 2:**
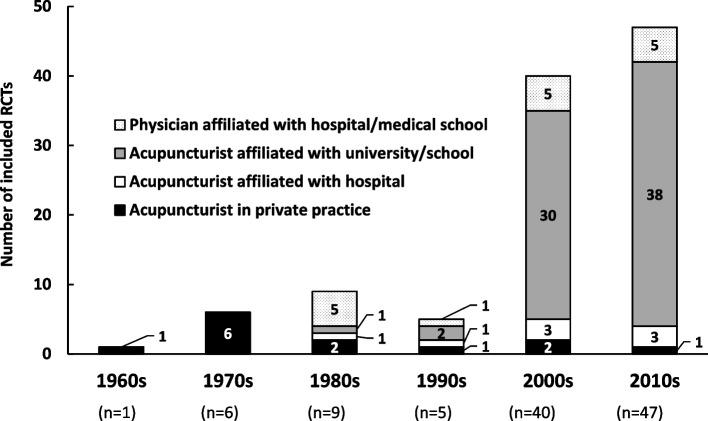
Qualifications and affiliations of the first author

Figure 2 depicts the first authors’ qualifications and affiliations stratified by decade. In the 1960s and 1970s, all the first authors were acupuncturists in private practice. In the 1980s, more than half of the first authors comprised one physician in medical school, but this was an exceptional case because one team published a series of auricular acupuncture trials. The proportion of acupuncturists in private practice decreased in the 1980s and 1990s, and declined further in and after the 2000s. Contrastingly, the proportion of authors who were professors, teachers, or students affiliated with a university or professional school increased remarkably in and after the 2000s.

**Fig. 3:**
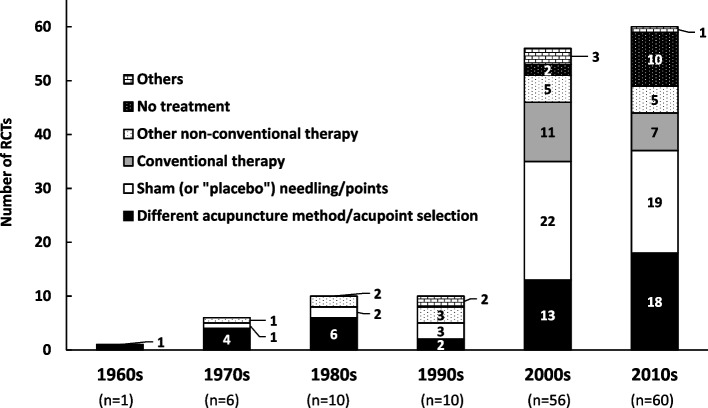
Type of control group. The number of some RCTs was duplicated because some trials had more than one control group (see Table 1)

Figure 3 shows the type of control group of the included RCTs stratified by decade. Before 1990, a different acupuncture method or different point selection (e.g., shallow vs. deep insertion, or manual needling vs. electroacupuncture) was the most dominant control setting. “Other non-conventional therapy” (e.g., transcutaneous electrical acupoint stimulation, laser acupuncture, or moxibustion) comprised 30% of the control group in the 1990s because one of five RCTs incorporated three different non-conventional treatment arms (3 divided by 10 arms: the actual number of the RCTs in this decade was 5). Sham (or “placebo”) needling and/or sham acupoints became the most dominant control treatment in the 2000s. Thereafter, in the 2010s, both types of sham needling/points and different acupuncture method/point selection became equally dominant, each accounting for approximately one-third.

**Fig. 4:**
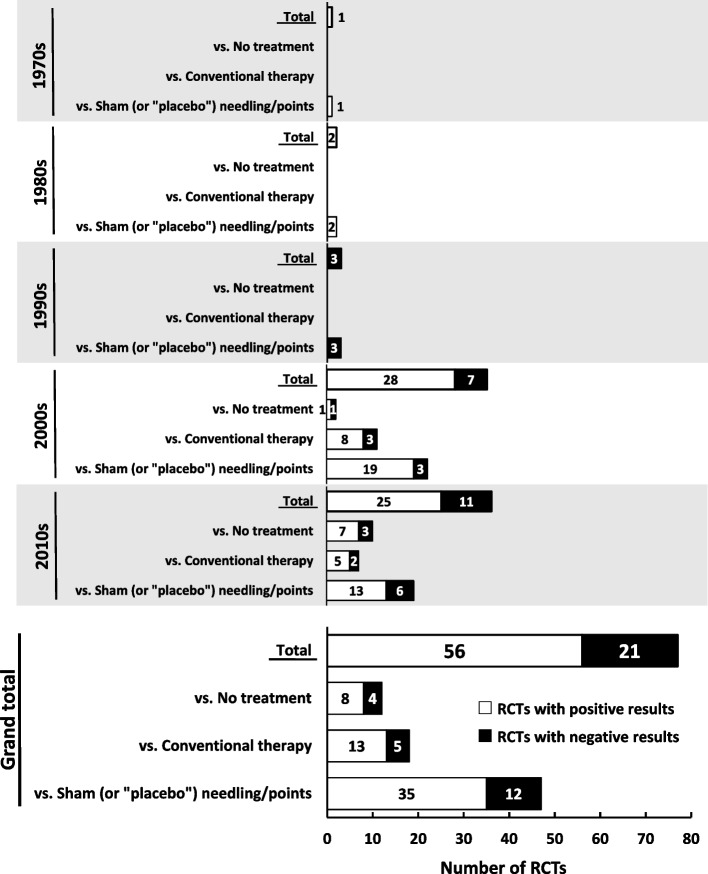
Proportion of positive RCTs stratified by decade. Only randomized controlled trials (RCTs) with control groups of sham/conventional/no treatment were included in this analysis. Some RCTs were counted more than once because they had more than one control group (see Table 1)

Figure 4 shows the proportion of RCTs with positive results with respect to the effect of acupuncture compared to sham needling/points, conventional therapy, or no treatment in the control group. In the 1990s, none of the three comparisons (actual number of RCTs: 2) revealed significantly superior effects of acupuncture. The first authors of these “negative” RCTs were an acupuncturist working at a university/school and a physician working at a hospital/medical school. In the 2000s, the first authors of negative studies (20%, 7 comparisons; actual number of RCTs, 7) were six acupuncturists working in a university/school and one acupuncturist working in a hospital. In the 2010s, the authors of negative studies (31%, 11 comparisons; actual number of RCTs, 11) were seven acupuncturists affiliated with a university/school, two physicians affiliated with a hospital/medical school, one acupuncturist affiliated with a hospital, and one acupuncturist in private practice. Three of these 11 RCTs were registered with a clinical trial registry site.

### RoB assessment of the included RCTs by decade

**Fig. 5:**
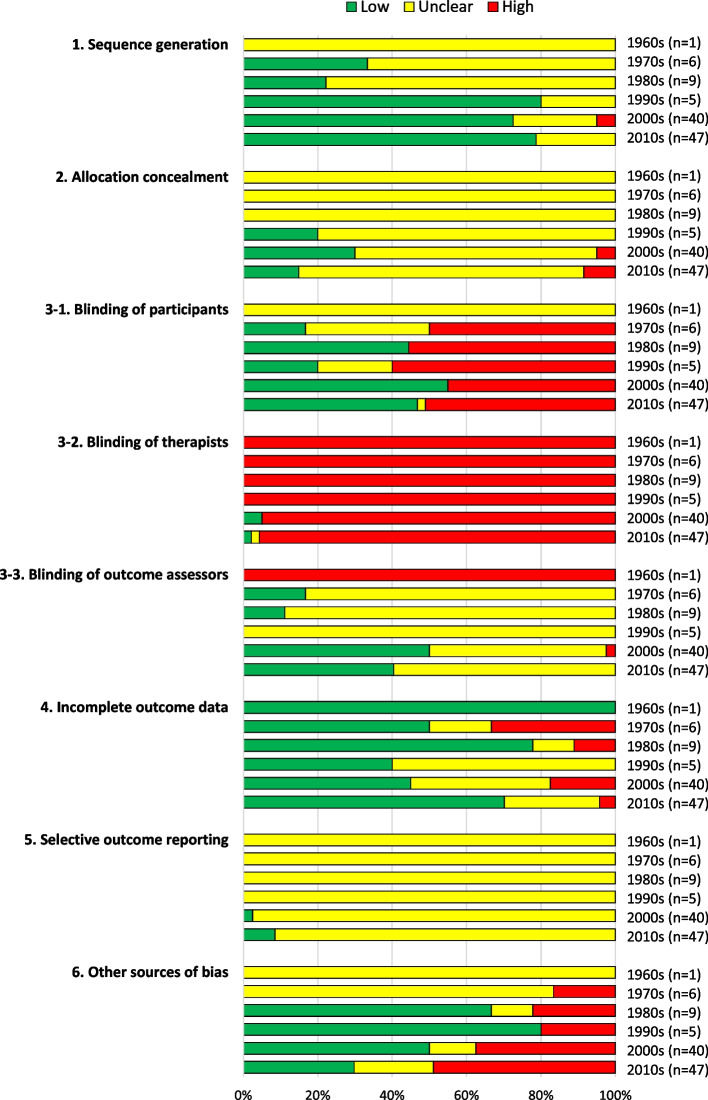
Risk of bias assessment

Figure 5 shows the aggregated RoB judgment of the RCTs stratified by decade. The RoB of the RCTs judged to be low with respect to “sequence generation” increased after the 1990s, accounting for 73%-80% of studies. Most RCTs were judged to have an unclear RoB with respect to “allocation concealment” because there was no description of this item, although 15%-30% of RCTs were judged to have a low RoB after the 1990s.

Overall, 45%-60% of the RCTs were judged to have a high RoB with respect to “blinding of participants,” except for RCTs published in the 1960s. Almost all included RCTs were judged to have a high RoB with respect to “blinding of therapists.” The exceptions were two (5%) RCTs published in the 2000s and one (2%) in the 2010s, which used the sham press tack needle as the control intervention. The proportion of RCTs judged to have a low RoB for “blinding of outcome assessors” increased in the 2000s (50%) and 2010s (40%), although the RoB of most other RCTs was judged to be unclear because of the lack of description of this aspect.

We exempted the 1960s from the assessment of “incomplete outcome data” because only one RCT was published in this decade; consequently, the proportion of RCTs with a low RoB for “incomplete outcome data” ranged between 40 and 78%. All RCTs were judged to have an unclear RoB for “selective outcome reporting,” due to the lack of published protocols for collation, except for one (3%) RCT in the 2000s and four (9%) in the 2010s. As the protocols of these five exceptional cases were published in a preceding paper or clinical trial registration platform on the internet, the reviewers could confirm that these trials were free of suggestion of selective outcome reporting. The reasons underlying the judgment of high RoB for “other sources of bias” included baseline imbalance, insufficient washout period in a crossover design, within-group comparisons, and excessive number of outcomes.

### Other indices related to the quality

Table 2 shows other indices suggestive of the quality or magnitude of the RCTs. The median sample size of the RCTs in each decade ranged between 20 and 41. None of the RCTs reported between the 1960s and 1980s clearly mentioned the acquisition of informed consent, whereas details about informed consent were stated in 93%-96% of RCTs published in and after the 2000s. Information on ethics committee approval was not mentioned in any of the RCTs before 2000. Information on clinical trial registration and registration number was reported only in the 2010s, although it was mentioned only in 9% of RCTs published in that decade. The proportion of adverse event reporting was the greatest in the 2000s, although it was limited to 33% of published RCTs.

**Table 2:** Other indices related to the quality of the RCTs included in this review

**Period** **(Decade)**	**Number of RCTs**	**Sample size**	**Informed consent**	**Ethics approval**	**Trial registration**	**Adverse events**
		**mean, median(SD)(min–max)**	**reported/not** **mentioned** **(% reported)**	**reported/not mentioned(% reported)**	**reported/not mentioned(% reported)**	**reported/not mentioned(% reported)**
1960s	1	32, 32 (N/A) (N/A)	0/1 (0%)	0/1 (0%)	0/1 (0%)	0/1 (0%)
1970s	6	22.7, 20 (6.9) (14–32)	0/6 (0%)	0/6 (0%)	0/6 (0%)	0/6 (0%)
1980s	9	37.4, 39 (14.3) (20–60)	0/9 (0%)	0/9 (0%)	0/9 (0%)	1/8 (10%)
1990s	5	93.0, 41 (103.0) (26–272)	2/3 (40%)	0/5 (0%)	0/5 (0%)	0/5 (0%)
2000s	40	36.8, 28 (31.3) (6–178)	37/3 (93%)	24/16 (60%)	0/40 (0%)	13/27 (33%)
2010s	47	36.1, 22 (41.5) (7–210)	45/2 (96%)	41/6 (87%)	4/43 (9%)	13/34 (28%)
Total	108	38.3, 29 (40.8) (6–272)	84/24 (78%)	65/43 (60%)	4/104 (4%)	27/81 (25%)

*RCT* Randomized controlled trial, *SD* Standard deviation, *N/A* Not available

## Discussion

### Authors’ affiliation and historical background

We have placed great emphasis on the qualifications and affiliations of the first author ever since our first systematic review [3]. This was because we thought that the status of the principal investigator would elucidate the historical background of acupuncture clinical research in Japan and would also be relevant to the selection of the control group. In the late 1960s and 1970s, a group of research-oriented practicing acupuncturists conducted controlled clinical trials in Japan under the guidance of Kosei Takahashi, a biostatistician affiliated with the University of Tokyo. The earliest RCT [14] included in the present systematic review was one of their achievements, which is probably the first parallel-armed RCT on acupuncture officially published worldwide [15]. The authors of the six RCTs published in 1970s were also directly or indirectly related to this group of practicing acupuncturists. However, the clinical research activities of these practicing acupuncturists do not seem to have extended to the entire group of acupuncturists in Japan because the proportion of practicing acupuncturists as first authors declined in the 1980s, never to rise again (Fig. 2).

On the other hand, the proportion of acupuncturists affiliated with a university or professional school rose in the 2000s and continued to rise further since then. This trend may be attributed to the dissemination of the concept of EBM throughout the healthcare community. Specifically, acupuncture professors and instructors who worked for universities or professional schools in Japan seemed motivated by the activity of a working group on clinical research methodology for acupuncture held in Kyoto [16], Tsukuba [17], and Aomori [18] for the publication of the WHO’s “Guidelines for Clinical Research on Acupuncture” in 1995 [19].

### Control group setting and sham needling

Before the 1990s, RCTs comparing different acupuncture methods or acupoint selections were dominant (Fig. 3) probably because most of the first authors (i.e., therapists in the present context) were acupuncturists in private practice (Fig. 2). It may have been difficult for practicing acupuncturists to assign their patients to the no-treatment or sham-treatment groups. We speculate that the number of RCTs with a sham-treatment arm increased after the 1990s owing to the increased number of researcher-acupuncturists working for or studying at a university or school. They had the option to establish sham or no-treatment groups, distinct from practicing acupuncturists.

In and after the 2000s, the proportion of RCTs comparing different acupuncture methods or acupoint selections increased again and nearly equaled that of RCTs comparing real and sham acupuncture treatment in the 2010s. RCTs with no-treatment and conventional therapy arms also increased in these two decades. One possible explanation for these changes is that Japanese acupuncture researchers had recognized the non-inertness of sham needling [20–22]. Japanese acupuncture researchers may have found it difficult to detect the real effect size of acupuncture through RCTs with a sham-treatment arm, especially because the stimulation of Japanese-style acupuncture is relatively weaker than that of Traditional Chinese Medicine-style acupuncture [23–25]. The lack of an optimal methodology to overcome this issue may be one of the background reasons why RCTs with various types of control groups have been conducted since the 2000s.

### Publication of negative RCTs

The frequency of distribution of published RCTs that found no significant difference between acupuncture and sham needling/points, conventional therapy, or no treatment was as follows: 100% in the 1990s, 20% in the 2000s, and 31% in the 2010s (Fig. 4). A systematic review published in 1998 reported that all acupuncture trials originating from China, Japan and Taiwan yielded positive results, suggesting the existence of publication bias [26]. Five acupuncture trials from Japan, which were retrieved through Medline, were published between 1991 and 1996. However, we could not locate any RCT on acupuncture for patients in Japan published in English using PubMed before 2000, at least according to our inclusion and exclusion criteria.

Given the present results (Fig. 4), it seems possible that the culture of submitting negative trial reports was already prevalent domestically to some degree, if not completely, in the Japanese acupuncture research milieu in the 1990s. However, this does not necessarily imply that Japanese acupuncture researchers had already developed research integrity, but rather that the professors and teachers working in universities or professional schools may have simply been driven by the urgency to publish more research papers. Our speculation is supported by the fact that 23 duplicate publications had to be eliminated in the literature screening process for the current review (Fig. 1).

### Quality of the RCTs with respect to the RoB (Fig. 5)

Although sequence generation in most RCTs published in and after 1990s was judged to have been performed properly, allocation concealment was not described in most reports. We suspect that the importance of performing and reporting allocation concealment was not well-recognized among acupuncture researchers even in the late 2010s.

It is inherently difficult to blind therapists to allocation in RCTs on acupuncture, except for trials with a sham press tack needle and those with a double-blind sham device [27] under limited conditions of needling. Hence, a high RoB is inevitable with respect to blinding of therapists. Blinding of participants is also difficult in the above-mentioned pragmatic trial design incorporating active or no-treatment control groups. Therefore, it is essential to blind the outcome assessors in RCTs on acupuncture. Nevertheless, we found that this has not been performed or reported sufficiently even in RCTs published in and after the 2000s.

The low RoB for incomplete outcome data does not necessarily mean that the data of drop-outs were handled appropriately. For example, there were no drop-outs in 22 of 47 RCTs conducted in the 2010s probably because of the short study duration, which consequently resulted in no incomplete outcome data. Only four (9%) RCTs were registered even in the 2010s (Table 2), which made the judgment of RoB for selective outcome reporting “unclear.”

One study assessed the trends in the RoB in RCTs on acupuncture in the last five decades, based on a search of the PubMed, Embase and three Chinese databases [2]. According to data derived from 368 systematic reviews, including 4,715 RCTs on acupuncture, there was a statistically significant reduction in the proportion of RCTs with unclear risk for random sequence generation and selective reporting, and a significant uptick in the trend of RCTs with unclear risk for blinding of participants and personnel, blinding of outcome assessment, and incomplete outcome data. The comparison of these data with ours revealed that only the declining trend in sequence generation is consistent. The proportion of unclear RoB in selective reporting in Japanese RCTs remains high because most protocols were not published beforehand.

### Quality of the RCTs from viewpoints of other indices

As seen in Table 2, there has not been much increase in the sample size of the RCTs even in this century. To date, few active plans are available for multicenter and/or multidisciplinary clinical trial projects in the Japanese acupuncture research community. We opine that a change in attitude is necessary to facilitate inter-institutional and interprofessional collaboration to expand the scale of their trials.

Recognition of the need to obtain and describe informed consent and ethics approval seems to have taken hold among Japanese acupuncture researchers in the 1990s and 2000s, respectively. On the other hand, the importance of trial registration and adverse event reporting was apparently not well-recognized even in the 2010s. This inadequacy may be partly attributed to the quality of the domestic Japanese journals that published the reports. A simple reminder that submitted manuscripts of RCTs should include information on trial registration and adverse events in the instructions to authors would improve quality. Moreover, there might be room for improvement in the rigor of peer review in domestic journals in the field of acupuncture.

### Limitations of the study

Since we excluded RCTs that were reported only as conference abstracts, it is not possible to determine the trends in all Japanese RCTs conducted on acupuncture for patients. In 2015, we collected 145 relevant RCT reports consisting of 86 full-length papers and 59 conference abstracts. Thus, we estimated that 70–80 relevant RCTs were reported as only conference abstracts, in addition to the 108 RCTs published as full-length papers. It is difficult to extract data on the study quality from conference abstracts, especially regarding RoB; hence, it was not realistic to include them in the present study. Shikura et al. reported that only 30 (31.3%) of 96 controlled trials (59 with healthy participants and 37 with patients) presented at annual meetings of the Japan Society of Acupuncture and Moxibustion between 2006 and 2010 had been published as full-length papers or proceedings [28]. Moreover, they found that 50% of these 96 trials did not find significant differences in the results. Since 80% of RCTs included in the present review that were published in the 2000s reported positive results (Fig. 4), it is likely that publication bias also affected the entire aspect of assessment in the present study. Nevertheless, we believe that our study is still informative and adequately reflects the state of acupuncture RCTs, so long as we refer to the relative changes within the obtained data from decade to decade.

It is reported that most RoB assessments for the same RCTs on acupuncture were moderately or substantially inconsistent among different systematic reviews [29]. This finding suggests that inter-study reliability is not sufficiently high for the assessment of RoB by systematic reviews on clinical acupuncture research. Although the present systematic review may also suffer from this issue, we believe that at least the within-study consistency is maintained because the same reviewers were involved in the RoB assessment throughout the study.

Apropos the quality of reporting with respect to the Consolidated Standards of Reporting Trials (CONSORT) statement [30] and its official extension, the Revised STandards for Reporting Interventions in Clinical Trials of Acupuncture (STRICTA) [31], a recent study reported that the mean compliance rate of 17 papers reporting RCTs on acupuncture published in the Journal of the Japan Society of Acupuncture and Moxibustion after the establishment of STRICTA was 75.4% (SD 13.5) [32]. We endeavor to conduct future studies focusing on the assessment of the quality of reporting of all RCTs on acupuncture published in Japan with respect to compliance with the CONSORT and STRICTA statements.

## Conclusions

This systematic review of RCTs on acupuncture for patients in Japan before 2020 revealed that the number of relevant RCTs increased in each subsequent decade, but the scale of the trials lacked growth with respect to the sample size. Besides, many reports are difficult to access internationally because all included RCTs published before 2000 and two-third of those reported after 2000 were written in Japanese. There seems to have been a turning point in the 1990s, which was marked by the shift in trial conductors from acupuncturists in private practice to those in universities or schools, type of control from different acupuncture methods/acupoints to a variety of control groups, including sham needling/points and conventional care, reporting of negative trials, and obtaining/reporting informed consent. These shifts probably arose from the changes in the circumstances surrounding acupuncture researchers, such as the dissemination of the concept of EBM and guidelines on clinical research.

The quality of the included RCTs, as assessed using the Cochrane RoB tool, has not necessarily improved over the decades, except for “sequence generation.” Although studies started describing ethics approval and adverse events in the 2000s, the overall reporting rate is still not sufficient. There is an urgent need to improve trial registration and adverse event reporting substantially. Furthermore, the need to exclude approximately 20% of reports of inappropriate duplicate publication (of the total number of RCT reports ultimately included) suggests insufficient dissemination of research ethics among the relevant researchers. Future clinical researchers of acupuncture in Japan should be mindful of these aspects to improve the quality of RCTs and publish them in a manner compliant with research ethics. Moreover, future researchers conducting systematic reviews, who wish to include the Japanese literature, need to take these issues into account.

## Supplementary Information

**Additional file 1. **Search strategy.

**Additional file 2. **List of all articles reporting the included RCTs.

## Data Availability

The datasets used and/or analyzed during the current study are available from the corresponding author on reasonable request.

## Abbreviations

CENTRAL: Cochrane Central Register of Controlled Trials
CONSORT: Consolidated Standards of Reporting Trials
EBM: Evidence-based medicine
PRISMA: Preferred Reporting Items for Systematic Reviews and Meta-Analyses
PROSPERO: International Prospective Register of Systematic Reviews
RCT: Randomized controlled trial
RoB: Risk of bias
STRICTA: STandards for Reporting Interventions in Clinical Trials of Acupuncture
