# Supplementary material for: Trends and quality of randomized controlled trials on acupuncture conducted in Japan by decade from the 1960s to the 2010s: a systematic review
Source: BMC Complement Med Ther. 2023 Mar 27;23:91. doi: 10.1186/s12906-023-03910-3 (PMC10041764; doi:10.1186/s12906-023-03910-3)
Supplement: Supplementary file 1 — Additional file 1. Search strategy. [file 12906_2023_3910_MOESM1_ESM.docx]

**Additional file 1: Search strategy**

**Ichushi Web (医中誌Web)**

#1 鍼療法/TH

#2 鍼灸療法/TH

#3 鍼灸医学/TH

#4 経絡/TH

#5 経穴/TH

#6 鍼/AL

#7 針療法AL/AL or はり療法/AL or 針治療/AL or はり治療/AL or or/AL and 針通電/AL or はり通電/AL or 経穴/AL or 経絡/AL

#8 臨床試験/TH

#9 クロスオーバー研究/TH

#10 臨床試験/AL or ランダム化臨床試験/AL or ランダム臨床試験/AL or 無作為化臨床試験/AL or 無作為臨床試験/AL or 比較試験/AL or ランダム化比較試験/AL or ランダム比較試験/AL or 無作為化比較試験/AL or 無作為比較試験/AL or ランダム化試験/AL or ランダム試験/AL and 無作為化試験/AL and 無作為試験/AL or RCT/AL

#11 クロスオーバー/AL

#12 #1 or #2 or #3 or #4 or #6 or #7

#13 (#12) and (RD=ランダム化比較試験,準ランダム化比較試験)

#14 #8 or #9 or #10 or #11

#15 #12 and #14

#16 #13 or #15

#17 (#16) and (PT=会議録除く CK=ヒト)

English translations

#1 acupuncture therapy/TH

#2 acupuncture and moxibustion therapy/TH

#3 acupuncture medicine/TH

#4 meridian/TH

#5 acupoint/TH

#6 acupuncture/AL

#7 Hari (acupuncture: Chinese character) therapy AL/AL or Hari (acupuncture: Japanese character) /AL or Hari (acupuncture: Chinese character) treatment /AL or Hari (acupuncture: Japanese character) treatment /AL or or/AL and electroacupuncture (Chinese character) /AL or electroacupuncture (Japanese character) /AL or acupoint /AL or meridian/AL

#8 clinical trial/TH

#9 cross-over study /TH

#10 clinical trial /AL or randomized controlled trial/AL or randomized clinical trial /AL or randomized (Chinese character) clinical trial /AL or randomization clinical trial/AL or controlled trial/AL or randomized comparative trial /AL or random controlled trial /AL or randomized controlled trial /AL or randomized (Chinese character) controlled trial /AL or randomized trial /AL or random trial /AL and randomized (Chinese character) trial /AL and random (Chinese character) trial/AL or RCT/AL

#11 cross-over/AL

#12 #1 or #2 or #3 or #4 or #6 or #7

#13 (#12) and (RD=randomized controlled trial, quasi-randomized controlled trial)

#14 #8 or #9 or #10 or #11

#15 #12 and #14

#16 #13 or #15

#17 (#16) and (PT=exclude conference abstract CK=human)

**Cochrane Central Register of Controlled Trials (CENTRAL)**

#1 MeSH descriptor: [Acupuncture Therapy] explode all trees

#2 MeSH descriptor: [Acupuncture] explode all trees

#3 MeSH descriptor: [Moxibustion] explode all trees

#4 MeSH descriptor: [Meridians] explode all trees

#5 MeSH descriptor: [Acupuncture Points] explode all trees

#6 MeSH descriptor: [Dry Needling] explode all trees

#7 acupunct*

#8 moxibust*

#9 moxa

#10 electroacupunct*

#11 dry needl*

#12 dry NEXT needl*

#13 acupoint*

#14 acupuncture point*

#15 #1 OR #2 OR #3 OR #4 OR #5 OR #6 OR #7 OR #8 OR #9 OR #10 OR #11 OR #12 OR #13 OR #14

#16 japan* OR nippon OR nihon

#17 #15 AND #16

in Trials (search limits)

**PubMed**

(("acupuncture therapy"[MeSH Terms] OR ("acupuncture"[MeSH Terms] OR "acupuncture therapy"[MeSH Terms]) OR "moxibustion"[MeSH Terms] OR "meridians"[MeSH Terms] OR "acupuncture points"[MeSH Terms] OR "acupunct*"[All Fields] OR "moxibust*"[All Fields] OR "moxa"[All Fields] OR "electroacupunct*"[All Fields] OR ("dry"[All Fields] AND "needl*"[All Fields]) OR "acupoint*"[All Fields] OR (("acupunctural"[All Fields] OR "acupuncture"[MeSH Terms] OR "acupuncture"[All Fields] OR "acupuncture therapy"[MeSH Terms] OR ("acupuncture"[All Fields] AND "therapy"[All Fields]) OR "acupuncture therapy"[All Fields] OR "acupuncture s"[All Fields] OR "acupunctured"[All Fields] OR "acupunctures"[All Fields] OR "acupuncturing"[All Fields]) AND "point*"[All Fields])) AND "Japan"[Affiliation]) AND ((randomizedcontrolledtrial[Filter]) AND (1000/1/1:2019/12/31[pdat]))

“Moxibustion” was included as a search term because we did not wish to miss RCTs of acupuncture that did not use the word “acupuncture” in the title and abstract, for example: moxibustion with warming needle (灸頭鍼).
