# Supplementary material for: Trends and quality of randomized controlled trials on acupuncture conducted in Japan by decade from the 1960s to the 2010s: a systematic review
Source: BMC Complement Med Ther. 2023 Mar 27;23:91. doi: 10.1186/s12906-023-03910-3 (PMC10041764; doi:10.1186/s12906-023-03910-3)
Supplement: Supplementary file 2 — Additional file 2. List of all articles reporting the included RCTs. [file 12906_2023_3910_MOESM2_ESM.docx]

**Additional file 2: List of all articles reporting the included RCTs**

English titles in parentheses “( )” are the official translations of the Japanese title by the article authors, and those in square brackets “[ ]” are the translations made by us due to the lack of an official English translations from the article authors.

**1960s**

木下晴都. 坐骨神経痛と針灸. 横須賀: 医道の日本社; 1969: p.136–8. (Kinoshita H. Acupuncture and moxibustion for the relief of sciatica. Yokosuka: Ido–no–Nippon–Sha; 1969: p.136–8)

**1970s**

出端昭男. 洞刺の降圧作用について. 日本鍼灸治療学会雑誌. 1970;19(2):26–8. (Debata A. Utilization of “Do–shi” (puncture of the carotid sinus) to reduce the blood pressure. The Journal of the Japan Acupuncture & Moxibustion Society. 1970;19(2):26–8)

木下晴都. 五十肩に対する特殊治療の効用. 日本鍼灸治療学会雑誌. 1973;22(1):23–8. (Kinoshita H. Effect of acupuncture on scapulohumeral periarthritis. The Journal of the Japan Acupuncture & Moxibustion Society. 1973;22(1):23–8)

倉本素宏. 椎間板ヘルニアに対する針通電の臨床的研究. 日本鍼灸治療学会雑誌. 1977; 26(2):45–8. (Kuramoto S. A clinical study of the effects of electrical acupuncture on protrusions of the intervertebral discs. The Journal of the Japan Acupuncture & Moxibustion Society. 1977;26(2):45–8)

木下晴都. 頸腕症候群に対する傍神経刺の臨床的研究. 日本鍼灸治療学会雑誌. 1978; 27(1):61–71. (Kinoshita H. Clinical observation of paraneural acupuncture for cervico–brachial syndrome. The Journal of the Japan Acupuncture & Moxibustion Society. 1978; 27(1):61–71)

黒須幸男. 腰痛に対する鍼灸治療(Ⅱ). 日本鍼灸治療学会雑誌. 1979;28(2):31–4. (Kurosu Y. Acupuncture and moxibustion for lumbago (II) – Comparative experiment of the therapeutic effectiveness of acupuncture and garlic moxibustion –. The Journal of the Japan Acupuncture & Moxibustion Society. 1979;28(2):31–4)

**1980s**

木下晴都, 木下典穂. 傍神経刺を坐骨神経痛に応用した臨床試験. 日本鍼灸治療学会雑誌. 1981;30(1):4–13. (Kinoshita H, Kinoshita N. Clinical research in the use of paraneural acupuncture for sciatica. The Journal of the Japan Acupuncture & Moxibustion Society. 1981;30(1):4–13.)

向野義人. 肥満の耳針療法－有効性及びその作用機序についての検討－. 全日本鍼灸学会雑誌. 1981;31(1):67–74. (Mukaino Y. Acupuncture therapy for obesity using ear needle treatment - analysis of effectiveness and mechanism. Journal of the Japan Society of Acupuncture and Moxibustion. 1981;31(1):67–74)

七堂利幸, 有地滋, 森悦子, 森俊豪, 鴫原丈一, 米山義. 不定愁訴に対する針灸効果－比較試験－. 全日本鍼灸学会雑誌. 1982;32(1):33–43. (Shichido T, Arichi S, Mori E, Mori S, Shigihara J, Yoneyama T. The effect of acupuncture on unidentified syndrome—sequential medical trial—. Journal of the Japan Society of Acupuncture and Moxibustion. 1982;32(1):33–43)

向野義人, 恒矢保雄, 服部徹. 肥満の耳針療法(2)－皮電点の意義について－. 全日本鍼灸学会雑誌. 1983;32(3):226–32. (Mukaino Y, Tsuneya Y, Hattori T. Auricular acupuncture for obesity—Concerning the significance of dermal points—. Journal of the Japan Society of Acupuncture and Moxibustion. 1983;32(3):226–32.)

向野義人, 荒川規矩男, 恒矢保雄. 肥満の耳針療法における噴門点と肺点の効果差. 全日本鍼灸学会雑誌. 1984;33(3): 279–84. (Mukaino Y, Arakawa K, Tsuneya Y. Comparison between cardia point and lung point on auricular acupuncture. Journal of the Japan Society of Acupuncture and Moxibustion. 1984;33(3): 279–84)

向野義人, 荒川規矩男. 肥満の耳針療法における味覚の変化. 全日本鍼灸学会雑誌. 1985; 34(3,4):211–16. (Mukaino Y, Arakawa K. Change of taste by ear acupuncture in simple obese patients. Journal of the Japan Society of Acupuncture and Moxibustion. 1985; 34(3,4):211–16.)

河内明, 豊田住江, 酒井佳, 北出利勝, 兵頭正義. 音楽リズム低周波置鍼療法についての検討. 全日本鍼灸学会雑誌. 1988;38(3):295–9. (Kawachi A, Toyoda S, Sakai Y, Hyodo M. An evaluation of music-rhythm low-frequency electro-acupuncture. Journal of the Japan Society of Acupuncture and Moxibustion. 1988;38(3):295–9)

藤抜龍治. 腰部脊柱管狭窄症の鍼灸治療に関する研究(2). 医道の日本. 1989;48(11):6–13. [Fujinuki R. Study on acupuncture and moxibustion for lumber spinal canal stenosis (2).

Ido no Nippon (The Japanese Journal of Acupuncture & Manual Therapies). 1989;48(11):6–13]

**1990s**

河内明, 角崎憲一, 篠原理恵, 井上琢磨, 豊田住江, 北出利勝, 他. 音楽リズム振動ベッド(BODYSONICPAD®)を併用した低周波置鍼療法の効果について. 全日本鍼灸学会雑誌. 1992;42(2):169–73. (Kawachi A, Kakuzaki K, Shinohara R, Inoue T, Toyota S, Kitade T, et al. Effectiveness of low frequency in situ needle acupuncture combined with Body Sonic Pad Therapy. Journal of the Japan Society of Acupuncture and Moxibustion. 1992;42(2):169–73)

佐々木和郎, 鍋田智之. 肩こり班・基礎１班合同ワークショップ報告：無作為化比較試験とインフォームド・コンセント. 全日本鍼灸学会雑誌. 1997;47(3):156–61. (Sasaki K, Nabeta T. Report on the Joint Workshop of the Shoulder Stiffness Group and Basic Group 1: Randomized controlled clinical trial and informed consent. Journal of the Japan Society of Acupuncture and Moxibustion. 1997;47(3):156–61)

鍋田智之, 古田高征, 北小路博司, 川喜田健司. 頚部コリ感に対する鍼刺激効果の臨床試験の試み. 全日本鍼灸学会雑誌. 1997;47(3):173–81. (Nabeta T, Furuta T, Kitakouji H, Kawakita K. Randomized controlled pilot study of acupuncture on neck stiffness. Journal of the Japan Society of Acupuncture and Moxibustion. 1997;47(3):173–81)

皆川宗徳, 石神龍代, 堀茂, 田中法一, 中村弘典, 河瀬美之, 他. 排尿障害に対する封筒法による臨床比較試験－中極穴の有効性について－. 全日本鍼灸学会雑誌. 1999;49(3)383–91. (Minagawa M, Ishigami T, Hori S, Tanaka N, Nakamura H, Kawase Y, et al. Controlled clinical trials using the envelope method for urinary dysfunction—the effectiveness of the zhongji (CV-3) —. Journal of the Japan Society of Acupuncture and Moxibustion. 1999;49(3)383–91)

西澤芳男, 永野富美代, 伏木信次, 西澤恭子, 吉岡二三. 大学生球技者外傷障害の治療とリハビリテーションに対する鍼治療のdouble blind testの検討. 関西臨床スポーツ医・科学研究会誌. 1999;8:57–9. [Nishizawa Y, Nagano F, Fushiki S, Nishizawa K, Yoshioka F. Double-blind test of acupuncture for treatment and rehabilitation of college ballplayers’ trauma/injury. Journal of Kansai Clinical Sports Medicine and Science. 1999;8:57–9]

**2000s**

松本勅, 川本正純, 伊藤幸治, 坂井友実. 第1期 腰痛に対する鍼の比較対照試験【他の保存療法との比較】平成7年度. In: 腰痛症に対する鍼治療の効果に関する研究. 東京: 東洋療法研修試験財団; 2000:5–15. [Matsumoto T, Kawamoto M, Ito K, Sakai T. [The 1st-stage controlled trial on acupuncture for low back pain【comparison with other conservative therapies】Fiscal year 1995. In: Study of acupuncture effects on low back pain. Tokyo: Public Interest Incorporated Foundation for Training and Licensure Examination in Anma–Massage–Acupressure, Acupuncture and Moxibustion; 2000:5–15]

Kitade T, Ohyabu H. Analgesic effects of acupuncture on pain after mandibular wisdom tooth extraction., Acupunct Electrother Res. 2000;25(2):109–15.

篠原昭二. 運動器系愁訴に対する経筋を応用した皮内刺鍼の有効性に関する臨床的研究. 明治鍼灸医学. 2000;26:65–80. (Shinohara S. Clinical effects of acupuncture (intradermal needles) based on the muscle meridians for the complaints on the joints and muscles during movements. The Bulletin of Meiji University of Oriental Medicine. 2000;26:65–80)

岡村由美子, 新井寧子, 荒牧元, 菊池尚子. 置針を併用した顔面神経麻痺の初期治療－続報－. Facial Nerve Research. 2000;20:123–5. (Okamura Y, Arai Y, Aramaki H, Kikuchi N. Original method of facial palsy treatment by acupuncture during intravenous drip infusion of ATP. Facial Nerve Research. 2000;20:123–5)

河瀬美之, 石神龍代, 堀茂, 中村弘典, 服部輝男, 田中法一, 他. 高血圧に対する足三里穴刺鍼の有効性について－封筒法による臨床比較試験－. 全日本鍼灸学会雑誌. 2000;50(2):185–9. (Kawase Y, Ishigami T, Hori S, Nakamura H, Hattori T, Tanaka N, et al. Effectiveness of the Zusanli (ST36) point for hypertension in acupuncture—Controlled clinical trials using the envelope method—. Journal of the Japan Society of Acupuncture and Moxibustion. 2000;50(2):185–9)

Kotani N, Hashimoto H, Sato Y, Sessler DI, Yoshioka H, Kitayama M, et al. Preoperative intradermal acupuncture reduces postoperative pain, nausea and vomiting, analgesic requirement, and sympathoadrenal responses. Anesthesiology. 2001;95(2):349–56.

坂井友実, 津谷喜一郎, 津嘉山洋, 中村辰三, 池内隆治, 川本正純, 他. 腰痛に対する低周波鍼通電療法と経皮的電気刺激法の多施設ランダム化比較試験. 全日本鍼灸学会雑誌. 2001;51(2):175–84. (Sakai T. Tsutani K, Tsukayama H, Nakamura T, Ikeuchi T, Kawamoto M, et al. Multi-center randomized controlled trial of acupuncture with electric stimulation and acupuncture-like transcutaneous electrical nerve stimulation for lumbago. Journal of the Japan Society of Acupuncture and Moxibustion. 2001;51(2):175–84)

石丸圭荘, 咲田雅一. 手術後疼痛に対する鍼鎮痛の効果. 東洋医学とペインクリニック. 2002;32(1,2,3,4):10–8. (Ishimaru K, Sakita M. Effects of acupuncture analgesia on post operative pain. Oriental Medicine and the Pain Clinic. 2002;32(1,2,3,4):10–8)

小澤庸宏, 小川貴司, 中川仁, 古東司朗. 内側型変形性膝関節症に対する鍼治療効果について—RCTによる刺鍼群と偽鍼群(鍼管刺激群)の治療効果の比較. 鍼灸Osaka. 2002;18(4): 393–6. (Ozawa N, Ogawa T, Nakagawa J, Kotou S. The effects of acupuncture and moxibustion on interior benikular osteoarthritis. Osaka Journal of Clinical Acupuncture & Moxibustion. 2002;18(4): 393–6)

古屋英治, 名雪貴峰, 八亀真由美, 古海博子, 篠原隆三, 二村隆一, 他. 肩こりに及ぼす円皮鍼の効果－偽鍼を用いた比較試験－. 全日本鍼灸学会雑誌. 2002;52(5):553–61. (Furuya E, Nayuki T, Yakame M, Furuumi H, Shinohara R, Nimura R, et al. Effect of press tack needle treatment on shoulder stiffness. Journal of the Japan Society of Acupuncture and Moxibustion. 2002;52(5):553–61)

Nabeta T, Kawakita K. Relief of chronic neck and shoulder pain by manual acupuncture to tender points—a sham-controlled randomized trial. Complement Ther Med. 2002; 10(4):217–22.

Tsukayama H, Yamashita H, Amagai H, Tanno Y. Randomised controlled trial comparing the effectiveness of electroacupuncture and TENS for low back pain: a preliminary study for a pragmatic trial. Acupunct Med. 2002;20(4):175–80.

Kurono Y, Egawa M, Yano T, Shimoo K. The effect of acupuncture on the coronary arteries as evaluated by coronary angiography: a preliminary report. Am J Chin Med. 2002;30(2&3):387–96.

山本一彦, 三村俊英, 赤尾清剛 吉川信, 粕谷大智, 山口智. 関節リウマチに対する鍼灸治療の果たす役割－関節症状の改善とQOL向上について－. 全日本鍼灸学会雑誌. 2003;53(5):626–34. (Yamamoto K, Mimura T, Akao K, Kitsukawa M, Kasuya D, Yamaguchi S. Acupuncture and moxibustion treatment for Rheumatoid arthritis—Multi-center randomized controlled trial of acupuncture and moxibustion for rheumatoid arthritis—. Journal of the Japan Society of Acupuncture and Moxibustion. 2003;53(5):626–34)

勝見泰和, 糸井恵, 小嶋晃義, 高取良太, 戸谷祐樹, 平澤泰介, 他. 高齢者の慢性腰痛に対する阿是穴鍼療法. リハビリテーション医学. 2004;41(12):824–9. [Katsumi Y, Itoi M, Kojima A, Takatori R, Todani Y, Hirasawa Y, et al. A-shi point acupuncture for chronic low back pain in the elderly]. The Japanese Journal of Rehabilitation Medicine. 2004;41(12):824–9)

今井賢治, 田和宗徳, 田口玲奈, 笹岡知子, 伊藤和憲, 北小路博司. 肩こりに対する鍼治療の臨床効果に関する研究－ランダム化比較試験(RCT)による検討－, 明治鍼灸医学. 2005; 36:158–9. [Imai K, Tawa M, Taguchi R, Sasaoka T, Itoh K, Kitakoji H. Study on clinical effects of acupuncture for shoulder stiffness—randomized controlled trial—. The Bulletin of Meiji University of Oriental Medicine. 2005; 36:158–9]

伊藤和憲, 勝見泰和. 高齢者の慢性腰下肢痛に対する鍼治療の効果－トリガーポイント鍼治療の有用性に関する比較試験－. 全日本鍼灸学会雑誌. 2005;55(4):530–7. (Effect of acupuncture treatment on chronic low back pain with leg pain in aged patients—a controlled trial about short-term effects of trigger point acupuncture—. Journal of the Japan Society of Acupuncture and Moxibustion. 2005;55(4):530–7)

中村幹佑, 奥村江里, 佐藤涼子, 脇本加奈, 菅原之人. 円皮鍼と貼付用磁気治療器の筋緊張緩和に関する検討. 東洋療法学校協会学会誌. 2006;29:69–72. [Nakamura K, Okumura E, Kato R, Wakimoto K, Sugawara K. Investigation of muscle tension relief by press tack needle and stick-on magnetic treatment device. The Journal of Japan College Association of Oriental Medicine. 2006;29:69–72]

坂口俊二, 若山育郎, 津嘉山洋. 慢性腰痛症に対する皮内鍼治療臨床試験(探索的研究). 関西鍼灸大学紀要. 2006;3:20–25. (Sakaguchi S, Wakayama I, Tsukayama H. Clinical trial of spinex treatment for chronic low back pain—A research study for a pragmatic trial—. The Bulletin of Kansai College of Oriental Medicine. 2006;3:20–25)

伊藤和憲, 南波利宗, 西田麗代, 河本真, 越智秀樹, 北小路博司. 大学生の肩こり被験者を対象にしたトリガーポイント鍼治療の試み－肩こりに関するアンケート調査と鍼治療の効果に関する臨床試験－. 全日本鍼灸学会雑誌. 2006;56(2):150–7. (Itoh K, Nanba T, Nishida A, Kawamoto S, Ochi H, Kitakoji H. The effect of trigger point acupuncture treatment on chronic neck and shoulder pain—Questionnaire and acupuncture treatment for university students. Journal of the Japan Society of Acupuncture and Moxibustion. 2006;56(2):150–7)

河瀬美之, 石神龍代, 中村弘典, 服部輝男, 皆川宗徳, 甲田久士, 他. 腰痛に対する鍼治療－偽鍼を対照群に用いた多施設ランダム化比較試験－. 全日本鍼灸学会雑誌. 2006;56(2):140–9. (Kawase Y, Ishigami T, Nakamura H, Hattori T, Minagawa M, Kouda H, et al. Acupuncture treatment for lower back pain—Multi-center randomized controlled trial using sham acupuncture as a control. Journal of the Japan Society of Acupuncture and Moxibustion. 2006;56(2):140–9)

Inoue M, Kitakoji H, Ishizaki N, Tawa M, Yano T, Katusmi Y, et al. Relief of low back pain immediately after acupuncture treatment—a randomised, placebo controlled trial. Acupunct Med. 2006;24(3):103–8.

Itoh K, Katsumi Y, Hirota S, Kitakoji H. Effects of trigger point acupuncture on chronic low back pain in elderly patients—a sham-controlled randomised trial. Acupunct Med. 2006;24(1):5–12.

伊藤和憲. 運動器疾患に伴う慢性疼痛に対する保存療法の意義－変形性膝関節症に対するTENSと鍼治療の効果－. 慢性疼痛. 2007;26(1):143–8. [Itoh K. Significance of conservative therapy for chronic pain associated with musculoskeletal disease—Effect of TENS and acupuncture for osteoarthritis of the knee—. The Journal of the Japanese Society for the Study of Chronic Pain. 2007;26(1):143–8]

廣田里子, 伊藤和憲, 勝見泰和. 高齢者の慢性腰痛患者に対するトリガーポイント鍼治療の試み－同一筋上に存在するトリガーポイントと圧痛点の刺激効果の違いについて－. 明治鍼灸医学. 2007;38:19–26. (Hirota S, Itoh K, Katsumi Y. Trigger point acupuncture treatment for chronic low back pain in elderly patients. The Bulletin of Meiji University of Oriental Medicine. 2007;38:19–26)

山本博司, 楳田高士, 吉備登, 増田研一. 変形性膝関節症に対するはり治療の臨床的効果　－無作為比較試験－. 関西医療大学紀要. 2007;1:86–9. [Yamamoto H, Umeda T, Kibi N, Masuda K. Clinical effects of acupuncture for osteoarthritis of the knee—a randomized controlled trial—. The Bulletin of Kansai University of Health Sciences. 2007;1:86–9]

坂口俊二, 金井成行, 戸田静男. ランダム化比較試験による冷え症に対する鍼灸治療の効果. 関西医療大学紀要. 2007;1:82–5. [Sakaguchi S, Kanai S, Toda S. Effect of acupuncture for Hiesho assessed by randomized controlled trial. The Bulletin of Kansai University of Health Sciences. 2007;1:82–5]

中島美和, 井上基浩, 糸井恵, 勝見泰和. ランダム化比較試験による頚肩部痛に対する鍼治療と局所注射の検討. 全日本鍼灸学会雑誌. 2007;57(4):491–500. (Nakajima M, Inoue M, Itoi M, Katsumi Y. A comparison of the effectiveness between acupuncture and local injection for neck pain: a randomized controlled trial. Journal of the Japan Society of Acupuncture and Moxibustion. 2007;57(4):491–500)

Itoh K, Katsumi Y, Hirota S, Kitakoji H. Randomised trial of trigger point acupuncture compared with other acupuncture for treatment of chronic neck pain. Complement Ther Med. 2007;15(3):172–9.

粕谷大智, 美根大介, 小糸康治, 杉田正道, 前野崇, 芳賀信彦. 骨格筋に対する鍼通電刺激のインスリン抵抗性に及ぼす影響. 現代鍼灸学. 2008;8(1):9–19. [Kasuya D, Mine D, Koito Y, Sugita M, Maeno T, Haga N. Influence of electroacupuncture stimulation of skeletal muscle on insulin resistance. Journal of the Japan Society of Modern Acupuncture and Moxibustion Research. 2008;8(1):9–19]

福野梓, 鶴浩幸, 片岡圭介, 山田潤. 鍼刺激による屈折変化非依存性の視力向上効果. 全日本鍼灸学会雑誌. 2008;58(2):195–202. (Fukuno A, Tsuru H, Kataoka K, Yamada J. Acupuncture stimulation improves visual acuity without refractive change. Journal of the Japan Society of Acupuncture and Moxibustion. 2008;58(2):195–202)

Itoh K, Hirota S, Katsumi Y, Ochi H, Kitakoji H. Trigger point acupuncture for treatment of knee osteoarthritis—a preliminary RCT for a pragmatic trial. Acupunct Med. 2008; 26(1):17–26.

宮本直, 伊藤和憲, 越智秀樹, 山田充彦, 大橋鈴世, 糸井恵. 変形性膝関節症に伴う痛みと運動機能に対する鍼治療の効果－鍼の刺入深度の違いによる治療効果の検討－. 全日本鍼灸学会雑誌. 2009;59(4):384–94. (Miyamoto T, Itoh K, Ochi H, Yamada M, Ohashi S, Itoi M. The efficacy of acupuncture treatment on pain and ability with osteoarthritis of the knee—Examination of the curative effect by the depth of an acupuncture needle—. Journal of the Japan Society of Acupuncture and Moxibustion. 2009;59(4):384–94)

伊藤里子, 伊藤和憲, 勝見泰和. ランダム化比較試験を用いた高齢者の慢性腰痛に対するトリガーポイント鍼治療の有用性の検討. 全日本鍼灸学会雑誌. 2009;59(1):13–21. (Itoh S, Itoh K, Katsumi Y. Effect of trigger point acupuncture treatment in older patients with chronic low back pain: randomized controlled trial. Journal of the Japan Society of Acupuncture and Moxibustion. 2009;59(1):13–21)

Inoue M, Hojo T, Nakajima M, Kitakoji H, Itoi M. Comparison of the effectiveness of acupuncture treatment and local anaesthetic injection for low back pain: a randomised controlled clinical trial. Acupunct Med. 2009;27(4):174–7.

Miyazaki S, Hagihara A, Kanda R, Mukaino Y, Nobutomo K. Applicability of press needles to a double-blind trial: a randomized, double-blind, placebo-controlled trial. Clin J Pain. 2009;25(5):438–44.

Itoh K, Itoh S, Katsumi Y, Kitakoji H. A pilot study on using acupuncture and transcutaneous electrical nerve stimulation to treat chronic non-specific low back pain. Complement Ther Clin Pract. 2009;15(1):22–5.

**2010s**

内村里恵, 岩川博文, 原田健一郎, 松尾卓, 池田奈津香, 村瀬健太郎, 他. 肩こりにおける柔軟性の変化－肩甲骨の動きを指標にして－. 東洋療法学校協会学会誌. 2010;33:222–7. [Uchimura R, Iwakawa H, Harada K, Matsuo T, Ikeda N, Murase K, et al. Change in flexibility in shoulder stiffness (*katakori*)—using scapular movement as an indicator—. The Journal of Japan College Association of Oriental Medicine. 2010;33:222–7]

渡邉勝之, 篠原昭二. 強力反応点への鍼刺激の有効性に関する研究－ランダム化比較試験による臨床的有効性の検討－. 全日本鍼灸学会雑誌. 2010;60(1):74–83. (Watanabe K, Shinohara S. Clinical effectiveness of acupuncture applied to strongly reactive points: randomized controlled trial regarding clinical efficacy. Journal of the Japan Society of Acupuncture and Moxibustion. 2010;60(1):74–83)

山崎翼, 福田文彦, 石崎直人, 今西二郎, 矢野忠. 慢性疲労に対する鍼治療の臨床的有効性の検討. 日本未病システム学会雑誌. 2010;15(2):186–96. (Yamazaki T, Fukuda F, Ishizaki N, Imanishi J, Yano T. The effect of acupuncture on chronic fatigue in healthy subjects. The Journal of Japan Mibyou System Association. 2010;15(2):186–96)

浅井紗世, 伊藤和憲, 浅井福太郎, 今井賢治, 北小路博司. 大学生の顎関節症被験者に対する鍼治療の試み－顎機能に関するアンケート調査と鍼治療の効果に関する臨床試験－. 全日本鍼灸学会雑誌. 2010;60(4):728–36. (Asai S, Itoh K, Asai F, Imai K, Kitakoji H. Effect of acupuncture treatment on temporomandibular disorders—Questionnaire and acupuncture treatment for university students—. Journal of the Japan Society of Acupuncture and Moxibustion. 2010;60(4):728–36)

石丸圭荘, 澤田規. スポーツ障害(膝関節痛)に対するLLLTと鍼治療の併用効果. 日本レーザー治療学会誌. 2010;9(2):63–6. (Ishimaru K, Sawada T. Combination effect of LLLT and acupuncture treatment in sports disorder (knee pain). Nihon Laser Chiryou Gkkai Shi [Journal of Japan Laser Therapy Association]. 2010;9(2):63–6)

Itoh K, Kitakoji H. Effects of acupuncture to treat fibromyalgia: A preliminary randomised controlled trial. Chin Med. 2010;5:11.

鶴浩幸, 北小路博司. 鍼刺激が矯正視力と眼精疲労および心拍数に与える影響. 東方医学. 2011;26(4):11–6. (Tsuru H, Kitakoji H. Effects of acupuncture treatment to best corrected visual acuity asthenopia and heart rate. Eastern Medicine. 2011;26(4):11–6)

鶴浩幸, 木村達哉, 木坂祐太, 高桑一裕, 北小路博司. 視力と疲れ目に対する鍼刺激の効果. 東洋医学2011;17(2):1–5. [Tsuru H, Kimura T, Kisaka Y, Takakuwa K, Kitakoji H. Effects of acupuncture stimulation for visual acuity and asthenopia. Oriental Medicine. 2011;17(2):1–5]

稲葉明彦, 宮本直. 専門学校生の肩こり被験者を対象とした鍼治療の試み－肩こりに関するアンケート調査と鍼の刺入深度の違いによる治療効果の検討－. 東洋医学. 2011;17(2):41–5.

[Inaba A, Miyamoto T. Acupuncture for professional schools students’ shoulder stiffness (*katakori*)—Questionnaire survey on *katakori* and evaluation on the effects of different depth of needle insertion. Oriental Medicine. 2011;17(2):41–5]

藤本幸子, 井上基浩, 中島美和, 糸井恵. 腰痛に対する腰部への鍼の刺入深度の違いによる治療効果の相違－ランダム化比較試験－. 全日本鍼灸学会雑誌. 2011;61(3):208–17. (Fujimoto S, Inoue M, Nakajima M, Itoi M. Difference between therapeutic effects of deep and superficial acupuncture needle insertion for low back pain: a randomized controlled clinical trial. Journal of the Japan Society of Acupuncture and Moxibustion. 2011;61(3):208–17)

山本博司, 楳田高士, 吉備登, 増田研一, 近藤哲哉, 中吉隆之, 他. 変形性膝関節症に対するはり治療の臨床的効果4－無作為化比較試験－. 関西医療大学紀要. 2011;5:7–11. (Yamamoto H, Umeda T, Kibi N, Masuda K, Kondoh T, Nakayoshi T, et al. Clinical effects of acupuncture for osteoarthritis of the knee 4—A randomized controlled trial—. The Bulletin of Kansai University of Health Sciences. 2011;5:7–11)

水嶋丈雄. パーキンソン病に対する薬物治療と鍼灸治療併用療法についての治療成績－2群間のランダム化比較試験－. 日本東洋医学雑誌. 2011;62(6):691–4. (Mizushima T. Treatment results between matched pair of L-dopa medication treatment and acupuncture treatment combination on Parkinson disease—The randomized controlled trial between 2 groups—. Kampo Medicine. 2011;62(6):691–4)

鶴浩幸, 芝貴洋, 北小路博司. 手三里穴または光明穴への鍼刺激が視力と眼疲労に与える影響. 東方医学. 2011;27(3):1–7. (Tsuru H, Shiba T, Kitakoji H. The effects of acupuncture stimulation in LI10 or GB37 to visual acuity and visual fatigue. Eastern Medicine. 2011;27(3):1–7)

豊福伸幸. 気管支喘息に対する鍼治療の効果の検討－運動誘発性喘息を対象として－. 明治国際医療大学誌. 2011;5:13–24. (Toyofuku N. Effectiveness of acupuncture treatment for bronchial asthma, focusing on exercise-induced asthma. The Bulletin of Meiji University of Integrative Medicine. 2011;5:13–24.)

中島達哉, 梅村浩史, 網濱直輝, 南靖昌, 篠原良征, 建部陽嗣. 慢性頭痛に対する鍼治療効果の検討－刺鍼部位によるランダム化比較試験－. 東洋療法学校協会学会誌. 2011;35:34–6. [Nakajima T, Umemura H, Amihama N, Minami Y, Shinohara Y, Tatebe H. Evaluation of the effect of acupuncture for chronic headache—A randomized controlled trial comparing insertion sites. The Journal of Japan College Association of Oriental Medicine. 2011;35:34–6]

岩元英輔, 村瀬健太郎, 谷之口真知子, 本石希美. 鍼通電療法が脳卒中患者の血管弾性に与える影響. 全日本鍼灸学会雑誌. 2012;62(3):216–25. (Eisuke I, Murase K, Taninokuchi M, Motoishi N. Effects of electroacupuncture on the arterial stiffness in stroke patients. Journal of the Japan Society of Acupuncture and Moxibustion. 2012;62(3):216–25)

福田晋平, 江川雅人, 苗村健治. パーキンソン病に対する鍼治療の臨床効果に関する研究－ランダム化比較試験(RCT)による検討－. 明治国際医療大学誌. 2012;6:21–45. (Fukuda S, Egawa M, Namura K. The clinical effects of acupuncture in patients with Parkinson’s disease: A randomized controlled trial. The Bulletin of Meiji University of Integrative Medicine. 2012;6:21–45)

Mikashima Y, Takagi T, Tomatsu T, Horikoshi M, Ikari K, Momohara S. Efficacy of acupuncture during post-acute phase of rehabilitation after total knee arthroplasty. J Tradit Chin Med. 2012;32(4):545–8.

Suzuki M, Muro S, Ando Y, Omori T, Shiota T, Endo K, et al. A randomized, placebo-controlled trial of acupuncture in patients with chronic obstructive pulmonary disease (COPD): the COPD-acupuncture trial (CAT). Arch Intern Med. 2012;172(11):878–86.

Tachibana K, Ueki N, Uchida T, Koga H. Randomized comparison of the therapeutic effect of acupuncture, massage, and Tachibana-style-method on stiff shoulders by measuring muscle firmness, VAS, pulse, and blood pressure. Evid-Based Complement Alternat Med. 2012;2012:989705.

Itoh K, Asai S, Ohyabu H, Imai K, Kitakoji H. Effects of trigger point acupuncture treatment on temporomandibular disorders: a preliminary randomized clinical trial. J Acupunct Meridian Stud. 2012;5(2):57–62.

古瀬暢達, 鶴浩幸, 北小路博司. 合谷穴への鍼刺激が視力と眼疲労に及ぼす影響. 東方医学. 2013;28(4):17–23. (Furuse N, Tsuru H, Kitakoji H. The effects of acupuncture stimulation on LI 4 to visual acuity. Eastern Medicine. 2013;28(4):17–23)

佐藤信, 竹内裕子, 坪根光伸, 市川和代, 野本繁, 小沢聖香, 他. 眼精疲労に対する鍼治療の検討. 東洋療法学校協会学会誌. 2013;36:115–8. [Sato S, Takeuchi Y, Tsubone M, Ichikawa K, Nomoto S, Ozawa S, et al. Evaluation of acupuncture for asthenopia. The Journal of Japan College Association of Oriental Medicine. 2013;36:115–8]

岩元英輔. 鍼通電刺激と経皮的電気刺激が褥瘡の皮膚温に及ぼす影響. 日本褥瘡学会誌. 2013;15(2):99–104. (Iwamoto E. Effects of electroacupuncture and transcutaneous electrical nerve stimulation on skin temperature in pressure ulcers. Japanese Journal of Pressure Ulcers. 2013;15(2):99–104)

岩元英輔, 村瀬健太郎, 谷之口真知子, 本石希美. 褥瘡における通常治療・ケアと鍼通電療法の併用効果－局所鍼通電療法と遠隔鍼通電療法の比較－. 全日本鍼灸学会雑誌. 2013;63(3):176–85. (Iwamoto E, Murase K, Taninokuchi M, Motoishi N. Effects of combining electroacupuncture with conventional care for the treatment of pressure ulcers—Comparison of local electroacupuncture and remote electroacupuncture—. Journal of the Japan Society of Acupuncture and Moxibustion. 2013;63(3):176–85)

鶴浩幸, 皇甫泰明, 江川雅人, 北小路博司. 百会穴の毫鍼刺激または鍉鍼刺激が視力と眼疲労に及ぼす影響. 東方医学. 2014;29(4):7–13. (Tsuru H, Koho Y, Egawa M, Kitakoji H. The effects of filiform acupuncture needle or blunt acupuncture needle stimulation on GV 20 to visual acuity and visual fatigue. Eastern Medicine. 2014;29(4):7–13)

井上基浩, 中島美和, 山口成広, 北小路博司. 腰下肢症状に対する腰部傍脊柱部刺鍼の効果－ランダム化比較試験－. 日本統合医療学会誌. 2014;7(2):28–34. (Inoue M, Nakajima M, Yamaguchi S, Kitakoji H. Effect of acupuncture in the lumbar paravertebral region on low back pain and lower limb symptoms—a randomized controlled trial—. Japanese Journal of Integrative Medicine. 2014;7(2):28–34)

Kikuchi A, Seki T, Takayama S, Iwasaki K, Ishizuka S, Yaegashi N. Effect of press needles on swallowing reflex in older adults with cerebrovascular disease: a randomized double-blind controlled trial. J Am Geriatr Soc. 2014;62(12):2438–40.

Itoh K, Saito S, Sahara S, Naitoh Y, Imai K, Kitakoji H. Randomized trial of trigger point acupuncture treatment for chronic shoulder pain: a preliminary study. J Acupunct Meridian Stud. 2014;7(2):59–64.

山本小百合, 池上典子, 尾﨑朋文. 産後の下肢むくみに対する円皮鍼の効果について. 東洋医学とペインクリニック. 2015;44(2):48–53. (Yamamoto S, Ikegami N, Ozaki T. Effects of treatment using round-head subcutaneous needles on lower limb swelling after childbirth. Oriental Medicine and the Pain Clinic. 2015;44(2):48–53)

Nakajima M, Inoue M, Itoi M, Kitakoji H. Difference in clinical effect between deep and superficial acupuncture needle insertion for neck-shoulder pain: a randomized controlled clinical trial pilot study. The Journal of the Japanese Society of Balneology, Climatology and Physical Medicine. 2015;78(3):216–27.

宮下眞理子, 大内晃一, 武田淳史. 足浴と鍼の併用による痩身効果の検討. 東京医療学院大学紀要. 2015;3:53–69. (Miyashita M, Ouchi K, Takeda A. Effects of the combination therapy of foot bathing and acupuncture for body weight control. [The Bulletin of University of Tokyo Health Sciences]. 2015;3:53–69)

山崎翼, 佐藤万代, 木村啓作, 片山憲史, 矢野忠. 労働者の疲労に対する鍼治療の直後効果－ランダム化比較試験－. 日本未病システム学会雑誌. 2016;22(1):8–14. (Yamazaki T, Sato M, Kimura K, Katayama K, Yano T. The effect of acupuncture on fatigue in healthy workers: a randomized controlled trial. The Journal of Japan Mibyou System Association. 2016;22(1):8–14)

渡邉淳一, 岡浩一朗. 中高齢者の慢性膝痛に対する円皮鍼の有効性－鍼師と被験者をマスクしたランダム化比較試験－. 全日本鍼灸学会雑誌. 2016;66(2):80–9. (Watanabe J, Oka K. Effectiveness of press needle among middle-aged persons with chronic knee pain. Journal of the Japan Society of Acupuncture and Moxibustion. 2016;66(2):80–9)

坂口俊二, 森英俊, 宮嵜潤二, 古田高征, 百合邦子, 周防佐知江, 他. 成熟期女性の冷え症に対する鍼治療の有効性を検証する多施設共同ランダム化比較試験. 日本東洋医学雑誌. 2016;67(4):340–6. (Sakaguchi S, Mori H, Miyazaki J, Furuta T, Yuri K, Suoh S, et al. Effectiveness of acupuncture therapy on *Hiesho* (cold disorder) in maturate stage females: a multicenter, randomized, prospective, controlled trial. Kampo Medicine. 2016;67(4):340–6)

Matsumoto-Miyazaki J, Asano Y, Yonezawa S, Nomura Y, Ikegame Y, Aki T, et al. Acupuncture increases the excitability of the cortico-spinal system in patients with chronic disorders of consciousness following traumatic brain injury. J Altern Complement Med. 2016;22(11):887–94.

Matsumoto-Miyazaki J, Asano Y, Ikegame Y, Kawasaki T, Nomura Y, Shinoda J. Acupuncture reduces excitability of spinal motor neurons in patients with spastic muscle overactivity and chronic disorder of consciousness following traumatic brain injury. J Altern Complement Med. 2016;22(11):895–902.

Sakaino M, Itoi M, Egawa M. Effect of electroacupuncture treatment for itching and skin condition of patients with atopic dermatitis: a randomized controlled trial. The Journal of Japan Mibyou System Association. 2017;23(1):1–11.

鶴浩幸, 長谷川希, 佐藤万代, 山﨑翼, 福田晋平, 江川雅人. 鍼刺激がフリッカー値および唾液アミラーゼに与える影響. 日本統合医療学会誌. 2017;10(1):124–6. (Tsuru H, Hasegawa N, Sato M, Yamazaki T, Fukuda S, Egawa M. The effect of acupuncture on flicker fusion frequency and salivary amylase. Japanese Journal of Integrative Medicine. 2017;10(1):124–6)

松浦悠人, 藤本英樹, 向ありさ, 古賀義久, 安野富美子, 坂井友実. 肩こりに対する鍼治療が唾液コルチゾール動態に与える影響：予備的ランダム化比較試験. 全日本鍼灸学会雑誌. 2017;67(2):124–32. (Matsuura Y, Fujimoto H, Muko A, Koga Y, Yasuno F, Sakai T. Effects of acupuncture treatment on changes in salivary cortisol concentration in patients with neck pain—a pilot randomized controlled trial. Journal of the Japan Society of Acupuncture and Moxibustion. 2017;67(2):124–32)

辻内敬子, 小井土善彦, 形井秀一, 善方裕美. ランダム化比較試験による骨盤位に対する鍼灸治療の効果の検討. 全日本鍼灸学会雑誌. 2017;67(3):215–23. (Tsujiuchi K, Koido Y, Katai S, Yoshikata H. Investigation of the effectiveness of acupuncture and moxibustion therapy as a treatment for breech presentation by a randomized controlled trial. Journal of the Japan Society of Acupuncture and Moxibustion. 2017;67(3):215–23)

Aoyama N, Fujii O, Yamamoto T. Efficacy of parietal acupoint therapy: scalp acupuncture for neck/shoulder stiffness with related mood disturbance. Med Acupunct. 2017;29(6):383–9.

大﨑彩加, 今枝美和, 北小路博司, 糸井恵, 井上基浩. 肩こりに対する鍼の刺入深度の違いによる効果の相違－予備的ランダム化比較試験－. 全日本鍼灸学会雑誌. 2018;68(1):10–20. (Osaki A, Imaeda M, Kitakoji H, Itoi M, Inoue M. Differences between therapeutic effects of deep and superficial acupuncture needle insertion for shoulder stiffness: a pilot randomized controlled clinical trial. Journal of the Japan Society of Acupuncture and Moxibustion. 2018;68(1):10–20)

Mazda Y, Kikuchi T, Yoshimatsu A, Kato A, Nagashima S, Terui K. Acupuncture for reducing pruritus induced by intrathecal morphine at elective cesarean delivery: a placebo-controlled, randomized, double-blind trial. Int J Obstet Anesth. 2018;36:66-76.
